# Quality of Life and Clinical Evaluation of Calcaneoplasty with a Balloon System for Calcaneal Fracture at 5 Years of Follow-Up

**DOI:** 10.1155/2021/5530620

**Published:** 2021-06-08

**Authors:** Giuseppe Maccagnano, Giovanni Noia, Giuseppe Danilo Cassano, Antonio Luciano Sarni, Raffaele Quitadamo, Costantino Stigliani, Francesco Liuzza, Raffaele Vitiello, Vito Pesce

**Affiliations:** ^1^Orthopaedics Unit, Department of Clinical and Experimental Medicine, Faculty of Medicine and Surgery, University of Foggia, Policlinico Riuniti di Foggia, Foggia, Italy; ^2^Orthopaedics Unit, Department of Basic Medical Science, Neuroscience and Sensory Organs, Faculty of Medicine and Surgery, University of Bari, Policlinico di Bari, Bari, Italy; ^3^Fondazione Policlinico Universitario A. Gemelli IRCCS, Rome, Italy

## Abstract

Calcaneal fractures are a challenging clinical problem. Management of this type of injury remains controversial, especially in the context of intra-articular fractures. Surgical treatment with open reduction and internal synthesis (ORIF) is considered the standard treatment for CF, but it is associated with many complications. Several minimally invasive techniques such as balloon-assisted reduction, pin fixation, and tricalcium phosphate augmentation have been proposed to avoid the frequent and recurrent postoperative problems related to these fractures. We retrospectively examined 20 patients (mean age was 54.5), all undergoing minimally invasive calcaneoplasty surgery at our Department of Orthopaedics and Traumatology between 2012 and 2016. X-ray and CT scan were performed preoperatively and at 5 years of follow-up (57.9 ± 6 months). The American Orthopaedic Foot and Ankle Society (AOFAS) score was used for clinical examination, and the Short-Form (36) Health Survey (SF-36) score and Visual Analogue Scale (VAS) were used to assess the Health-Related Quality of Life (HRQoL). All 20 patients were available at the final follow-up. The mean AOFAS score was 82.25/100. The VAS results attest an overall average of 2.7/10 (0–9). The average of the parameters “Physical Health” and “Mental Health” was, respectively, 81.25 and 83.55. In terms of postoperative complications, we observed no cases of superficial or deep infections. Clinical response after balloon-assisted reduction, pin fixation, and tricalcium phosphate augmentation has shown a comparable or better outcome according to the AOFAS and VAS score. Quality-of-life scores, obtained according to the SF-36 questionnaire, are considered high. From both a clinical and quality-of-life point of view, our study highlights that there is not gender distinction. Further comparative studies with a higher number of patients are needed which assess the quality of life in the various techniques used to treat calcaneal fractures.

## 1. Introduction

Calcaneal fractures (CF) are common injuries of the lower extremity, accounting for 2% of all fractures and 60% of all tarsal bone fractures. [1] These types of fractures are generally caused by high-energy trauma, such as a fall from heights or a car incident, and may be extra-articular or intra-articular.

The economic impact of the injury is considerable because 80–90% of these fractures occur in men in the early years of employment. As a result, the injury negatively impacts them for several years after the injury and many are unable to return to their previous employment.

Clinically, the injuries manifest with swelling, pain (mainly located below the peroneal sheath), distal edema at the level of the calcaneus cuboid joint, and ecchymosis. Sometimes, a deformity of the anatomical profile of the hindfoot is noticeable, and vasculonervous lesions are rare [2].

Calcaneal fractures are a challenging clinical problem because of the complex anatomy of the calcaneus, frequent involvement of the subtalar joint, and frequent joint displacement.

The management of this type of injury remains controversial, especially in the context of intra-articular fractures [3]. In the past, conservative treatment for intra-articular CF was preferred, but nowadays, it should be used only in extra-articular fractures or selected cases [4].

The main goals of the treatment are to restore the congruence of the subtalar joint and to restore the calcaneal width, height, shape, and alignment, thus avoiding medial and lateral conflict and allowing the patient to resume a normal lifestyle [5].

Surgical treatment with open reduction and internal synthesis (ORIF) [6] is considered the standard treatment for CF, but it is associated with many complications, particularly in patients with systemic diseases, such as diabetes and peripheral vascular disease.

Therefore, several minimally invasive techniques have been proposed to avoid the frequent and recurrent postoperative problems related to these fractures, to ensure good reduction with fewer complications and to reduce preoperative and hospitalization times; these techniques include arthroscopically assisted reduction and fixation, external fixation, balloon calcaneoplasty [7, 8].

Various authors have described and compared the numerous surgical techniques and outcomes [9].

Preliminary results obtained with calcaneoplasty appear to be satisfactory.

There are several studies evaluating the outcomes of calcaneoplasty at 1-year follow-up [10] or long-term follow-up [11]. Although the clinical outcome is always analyzed, the quality of life is rarely evaluated.

The aim of this study is to evaluate at a 5-year follow-up patients treated with calcaneoplasty, using clinical scales such as the AOFAS and VAS and Quality-of-Life scale (SF-36).

## 2. Materials and Methods

We retrospectively examined a group of twenty patients who underwent calcaneoplasty with a Balloon System (Kyphon^®^, Medtronic, Minneapolis, MN, USA), Tricalcium phosfate augmentation, and pin fixation for a calcaneal fracture with thalamic articular involvement, at our Department of Orthopaedics and Traumatology between 2012 and 2016. Our study sample consisted of 12 males and 8 females with a mean age of 54.5 (ranging between 43 and 72).

The mean follow-up was 57.9 ± 6 months.

  Inclusion criteria were (a) age over 18 years and (b) calcaneal fracture (Sander's type II, III, and IV)
  Exclusion criteria were (a) patients with a positive history of previous, concomitant, or subsequent fractures or who underwent surgery of the affected lower extremity; (b) patients with pathologies that may affect foot function, such as lumbar radiculopathy, Achilles tendinitis, and Morton's neuroma; and (c) fracture with great displacement of the sustentacular part of the calcaneus body

In the preoperative phase were performed an X-ray and CT examination.

**Figure 1:**
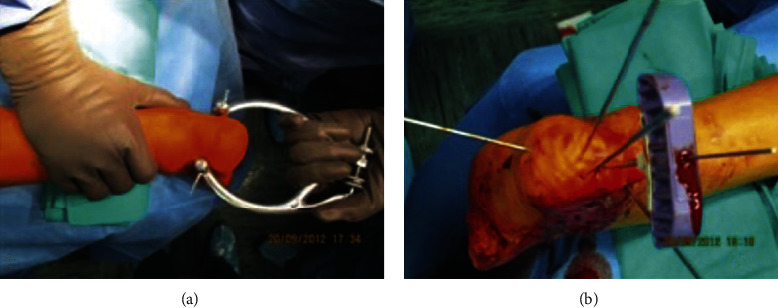
(a) Traction used to correct varus/valgus displacement. (b) Trocar insertion and K-wires positioned to maintain the reduction.

All cases underwent surgery in the prone position, with the lateral and AP/thalamic view of a double image intensifier control. Additional techniques of reduction were often used. Using a calcaneal traction, in fact, we managed to correct varus/valgus displacement (Figure 1(a)).

Fluoroscopy was used to determine the quality of calcaneal alignment and fracture reduction. K-wires parallel to the articular surface were applied to maintain the reduction. A trocar was placed into the calcaneus (Figure 1(b)), and then, a cannula followed by the insertion of a bone tamp was attached to a digital manometer.

The balloon was inflated under fluoroscopy.

**Figure 2:**
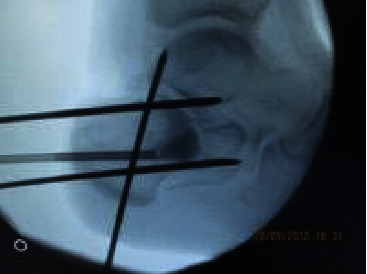
Injection of the bone cement. It is noted that the k-wires positioned distally as a palisade allow the balloon to reduce the subtalar joint.

Typically, the resulting reduction force of the expanding balloon was given by the aid of K-wires, positioned distally as a palisade (see Figure 2).

Additional K-wires were used to maintain the reduction obtained. Prior to its injection into the defect, bone cement (CaPO4) was prepared and the balloon was removed.

No cast was applied. In all cases, an X-ray was performed in the immediate postoperative period, which demonstrated good reduction of postfracture calcaneal varus and restoration of calcaneal volume with satisfactory reduction of the subastragalic joint surface.

Patients were discharged on average after 2 days. Percutaneous K-wires were removed on postoperative day 7.

All patients underwent the same rehabilitation protocol, with a progressive load from the 15^th^ day after surgery. Our study, thus, followed the impact of calcaneoplasty surgery over the years, identifying a midterm postoperative period at 5 years after surgery. At the follow up, the patient's condition in terms of foot biomechanics and quality of life was evaluated. Patients were subjected to

- history and objective examination according to the CRF (Case Report Form)
- X-ray investigation
- administration of questionnaires: (1) AOFAS (American Orthopaedic Foot and Ankle Society); (2) pain VAS (Visuo-Analogic Scale); and (3) SF-36 (Short-Form-36)

Data were recorded by two independent orthopedic surgeons (SAL and QR), and the values reported in Tables 1 and 2 are the average of the two measurements.

Student's *t*-test was used for statistical analysis of continuous variables, with values below 0.05 being considered significant.

## 3. Results

All 20 patients were available for clinical and radiographic follow-up at an average of 57.9 ± 6 months. The summary of results is shown in Tables 1 and 2.

At the last follow-up, the mean AOFAS score was 82.25/100. According to AOFAS score, clinical results were excellent in 6 (30%) cases, good in 7 (35%), fair in 4 (20%), and poor in 3 patients (15%). Male patients have reached an average score of 86.5/100 ± 14.5 and women 75.87/100 ± 9.51. No gender difference emerged (*p* value = 0.086).

The VAS results attest an overall average of 2.7/10 (0–9), an average from the male population of 2/10 and from the female population of 3.75/10.

At the last follow-up, SF-36 was used to evaluate patients' Health-Related Quality of Life (HRQoL). The average of Physical Health (PH) and Mental Health (MH) was 81.25 ± 14.35 for PH and 83.55 ± 7.63 for MH. Our analysis revealed no significant difference in mean scores for the physical component of SF-36 between male (86.18 ± 15.57) and female (73.85 ± 8.60), *p* value = 0.057. However, no gender difference emerged in the comparison of the mental component of the SF-36 (male 85.07 ± 6.85, female 81.28 ± 8.61, *p* value = 0.288).

Regarding intraoperative complications, we had 1 (5%) case of tricalcium phosphate migration at the subtalar joint. In terms of postoperative complications, we observed no cases of superficial or deep infections. Two (10%) patients suffered from plantar fasciitis, successfully treated with physical therapy.

## 4. Discussion

Our study is based on the evaluation of a recently introduced treatment, calcaneoplasty, focusing on the impact it has on the patient's quality of life. The ORIF technique is still widely adopted by orthopedic surgeons [12]. It is normally performed by the conventional L-shaped lateral method, even if the soft tissues covering the lateral calcaneal wall are extremely thin and fragile, which can lead to wound problems. As a consequence, complications following calcaneal fracture are common clinical problems which cannot often be prevented, especially in patients with comorbidities such as peripheral vascular disease, a smoking habit, and diabetes mellitus [13]. Percutaneous treatment with a balloon reduction device can enable surgeons to treat this lesion in the first days after trauma and without wound complications associated with ORIF. The reduction is obtained using k-wires and balloon inflating. Then, the cement injection guarantees a stable construct. Various forms of bone replacements, such as PMMA by Jacquot et al. [14], tricalcium phosphate by Labbe et al. [15] and Vicenti et al. [11], and calcium phosphate by Biggi et al. [16] or calcium sulfate by Gupta et al. [8], have been used for this function. Due to its multiple characteristics and properties, tricalcium phosphate was selected in the current study.

Patients were evaluated respecting a postoperative time of at least 5 years of follow-up (57.9 months ±6). For each of them, a biphasic analysis was chosen, in which the first step imposed a clinical evaluation and in the second step, we aimed to evaluate the quality of life.

In the current study, we observed good and excellent clinical results. The mean AOFAS and VAS scores were 82.25/100- grading “good” and VAS 2.7/10- grading “low pain/mild pain,” respectively. It is also important to emphasize the distinction in the AOFAS outcome between the two sexes, in favor of men with a difference of 10.62 points, but it has not statistical significance. Only 2 patients had a VAS greater than 5.

AOFAS scores presented by Chen et al. in their work are better (AOFAS 82.25 vs. 91.7) with respect to our result, and this may be due to the sample under examination since the patients analyzed by Chen et al. [17] had an average age of 31.1 years compared to that of our group in question (54.5). When we compare our results to the study of Vicenti et al. [11], they are comparable (AOFAS 82; mean age of the sample under examination 55.2).

In the study by Jacquot et al. [14], the mean AOFAS score was 84.5.

Focusing on the SF-36 evaluation, there are no studies that focus on the physical and psychological sphere of the patient treated with calcaneoplasty. Van Tetering et al. [18] show, using SF-36 scores, that patients with intra-articular calcaneus fractures had a worse quality of life than patients who had undergone surgery such as total hip or knee replacement or myocardial infarctions. Our scores of the SF-36 questionnaire are better if compared to those obtained in ORIF technique studies [19].

Our results show a good recovery both from a psychological and physical point of view, at a follow up of 5 years and do not show a gender difference in clinical results.

## 5. Conclusions

Although a small number of patients in our study, considering the recent introduction of balloon-assisted reduction, pin fixation and tricalcium phosphate augmentation for calcaneal fracture, have shown good clinical results, good outcomes in the PH and MH and an improvement in the clinical scores were observed. They were comparable and, in some cases, better than the most used ORIF (Open reduction and Internal Synthesis) which reported slightly lower AOFAS scores. Finally, both from a clinical and quality-of-life point of view, our study highlights that there is no gender distinction. Further comparative studies with a higher number of patients are needed which assess the quality of life in the various techniques used to treat calcaneal fractures.

## Figures and Tables

**Table 1:** AOFAS and VAS score.

Case no.	Gender	Age	Side	AOFAS	VAS
1	M	53	Right	53	9
2	M	66	Left	100	0
3	M	37	Right	84	2
4	M	50	Right	100	0
5	M	52	Left	84	1
6	M	74	Left	80	3
7	M	59	Right	84	0
8	M	34	Left	100	0
9	M	59	Right	100	0
10	M	45	Left	100	0
11	M	64	Left	73	6
12	M	43	Right	80	3
13	F	53	Right	65	2
14	F	66	Left	73	4
15	F	64	Right	61	5
16	F	74	Right	80	5
17	F	59	Left	77	5
18	F	43	Right	77	4
19	F	45	Left	84	4
20	F	50	Right	90	1
Average		54.5		82.25	2.7

**Table 2:** SF-36 scale.

Case no.	Gender	Age	PF	RP	BP	GH	VT	SF	RE	MH	Average PH	Average MH
1	M	53	60	50	36.6	50	66.6	70	83.33	69.88	49.15	72.45
2	M	66	100	100	100	80	74.9	100	100	74.9	95	87.45
3	M	37	100	100	82	75	74.9	100	100	74.9	89.25	87.45
4	M	50	100	100	100	80	74.9	100	100	74.9	95	87.45
5	M	52	100	100	100	80	70.75	90	100	76.6	95	84.34
6	M	74	100	100	82	75	87.55	100	100	90.04	89.25	94.4
7	M	59	100	100	100	80	70.75	90	100	76.6	95	84.34
8	M	34	100	100	100	80	70.75	90	100	76.6	95	84.34
9	M	59	100	100	100	80	70.75	90	100	76.6	95	84.34
10	M	45	100	100	82	75	74.9	100	100	74.9	89.25	87.45
11	M	64	100	100	82	75	87.55	100	100	90.04	89.25	94.4
12	M	43	76.6	60	40.6	55	66.6	70	83.33	69.88	58.05	72.45
13	F	53	63.3	100	30	60	58.1	40	100	59.76	63.32	64.46
14	F	66	100	87.5	82	80	74.9	90	83.33	87.45	87.37	83.92
15	F	64	76.6	100	50	60	58.1	70	100	59.76	71.65	71.96
16	F	74	96.6	75	54	65	79.15	90	100	76.6	72.65	86.44
17	F	59	63.3	87.5	65	70	74.9	80	83.33	87.45	71.45	81.42
18	F	43	80	65	50	60	79.15	90	100	76.6	63.75	86.44
19	F	45	100	75	82	80	79.15	90	100	87.45	84	89.15
20	F	50	96.6	75	65	70	79.15	90	100	76.6	76.65	86.44
Average		54.5	90.65	89.47	74.16	71.5	73.67	87	96.67	76.87	81.25	83.55

## Data Availability

Data are available upon request.
